# Subtomogram analysis: The sum of a tomogram’s particles reveals molecular structure *in situ*

**DOI:** 10.1016/j.yjsbx.2022.100063

**Published:** 2022-02-04

**Authors:** Friedrich Förster

**Affiliations:** Structural Biochemistry, Bijvoet Centre for Biomolecular Research, Utrecht University, Uni-versiteitsweg 99, 3584 CG Utrecht, the Netherlands

**Keywords:** Cryo-electron tomography, Subtomogram averaging, Image analysis, Correlation, Native structural biology

## Abstract

Cryo-electron tomography is uniquely suited to provide insights into the molecular architecture of cells and tissue in the native state. While frozen hydrated specimens tolerate sufficient electron doses to distinguish different types of particles in a tomogram, the accumulating beam damage does not allow resolving their detailed molecular structure individually. Statistical methods for subtomogram averaging and classification that coherently enhance the signal of particles corresponding to copies of the same type of macromolecular allow obtaining much higher resolution insights into macromolecules. Here, I review the developments in subtomogram analysis at Wolfgang Baumeister’s laboratory that make the dream of structural biology in the native cell become reality.

## Highlights

• Cryo-electron tomography depicts the biochemical machinery inside the cell at molecular detail.
• Subtomogram averaging overcomes the beam sensitivity of biological macromolecules and enables resolving macromolecules that occur in multiple copies at much higher resolution than the individual observations.
• Key developments of this approach were pursued in the laboratory of Wolfgang Baumeister.

## Graphical abstract

**Figure:**
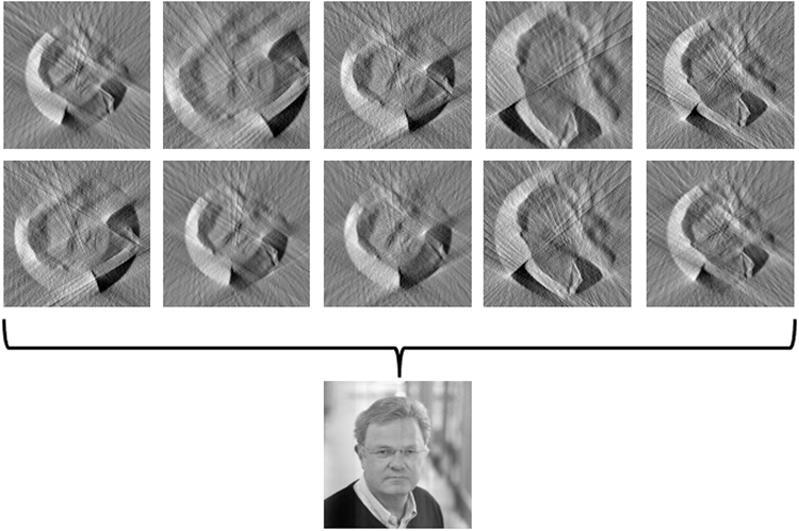

## Introduction

Electron microscopes have unique resolving power. Soon after electron microscopes had been developed, they were applied to study biological materials at fine detail, for example by Helmut Ruska, the brother of Nobel laureate Ernst Ruska. One of Helmut Ruska’s PhD students was Wolfgang Baumeister, who worked tirelessly to unleash the full potential of electrons to eventually enable a ‘voyage to the inner space of cells’ (Baumeister, 2005). I am honored to contribute to this special issue honoring the achievements of Wolfgang Baumeister in structural biology and to elaborate on one stage of this journey, which involved the development of subtomogram analysis.

The road from the first transmission electron microscopes (TEMs) to ‘visual proteomics’ of the cell has been long. The first obvious problem is that TEMs provide two-dimensional (2D) images, while the (biological) world is three-dimensional (3D). Due to the small wavelength of high-energy electrons and the resulting large depth of focus a TEM image is approximately a projection of the 3D sample. The mathematician Johann Radon had already shown that the 3D density can, in principle, be reconstructed from its projections in 1917 (Radon, 1917) and those principles were re-discovered in the 60 s in electron microscopy (DeRosier and Klug, 1968). While the seminal work of DeRosier and Klug exploited internal symmetries of molecules to access the different views required for 3D reconstruction, Hart suggested a procedure for 3D reconstruction of pleiomorphic objects such as cells (Hart, 1968): the polytropic montage involved collection of multiple TEM images of the sequentially tilted samples, which were then computationally merged. This process is now referred to as electron tomography (ET). Another early pioneer of ET has been the Max-Planck director Walter Hoppe (Hoppe, 1969), whose legacy in Martinsried was continued by Wolfgang Baumeister.

ET was initially applied to chemically fixed and dehydrated samples that had been embedded in resin and stained with heavy metal salts. These sample preparation methods have previously been used for the 2D imaging of ultrathin sections providing seminal insights into the ultrastructure of cells, for example into the secretory pathway (Palade, 1975). However, resin-embedded samples have a major limitation: dehydration removes many soluble components of the cell and distorts the molecular structure. With the advent of cryo-preparation, i.e., the vitrification of hydrated samples through ultra-fast freezing (Dubochet et al., 1988), this constraint was overcome. Whereas classical plastic preparation eventually left the researcher guessing which features of images were interpretable and which ones were artifacts of the preparation, the cryo-preparation method, in principle, allowed obtaining faithful images of the cell. Wolfgang Baumeister summarized this advantage as *In vivo veritas* (Plitzko et al., 2002).

Yet, the superior cryo-preparation comes at a price: not only is the entire sample preparation and imaging process an enormous engineering challenge because the sample must be kept below 140 K constantly and deposition of contaminating water vapor must be avoided, the cryo-sample is also extremely sensitive to the electron beam. Consequently, electron doses for imaging the samples must be low, typically not exceeding 100 e^-^/Å^2^, resulting in extremely noisy images (see also review by Frangakis in this issue). Development of automated ‘low dose’ data acquisition procedures, which minimize the dose spent on necessary microscope adjustments during data acquisition, were key to obtaining cryo-tomograms (Dierksen et al., 1993, Dierksen et al., 1992, Grimm et al., 1997, Grimm et al., 1998). Nevertheless, even using all tolerable electrons to record the data, before the electron damage itself obscures features, still results in images with very low signal-to-noise ratio (SNR). This beam sensitivity ultimately limits the resolution to a few nanometers with finer features being overwhelmed by the noise. This level of detail is typically insufficient for molecular interpretation leaving many structural biologists distinctly unimpressed.

The power of statistics and computation eventually helped get the structural biology community excited about cryo-ET. Most tomograms of cells, viruses or organelles contain multiple copies of the macromolecules of interest, which can be used to obtain higher resolution insights into the structures of these particles. Saxton and Baumeister developed a computational method called correlation averaging in the early 1980′s to compensate for imperfections in 2D crystals (Saxton and Baumeister, 1982). Precise alignment of individual particle images in the computer results in averages, where the signal of the contributing images is coherently amplified similar as in a well-ordered crystal, increasing the resolution substantially. This principle can be extended to particles without any near-range order in a 3D cell. The summed signal of precisely aligned subtomograms depicting the same macromolecule reveals more statistically significant information than each individual subtomogram does (Förster and Hegerl, 2007).

In this personal recollection I summarize the problem of subtomogram averaging and classification, the beginnings of this approach in Wolfgang Baumeister’s lab and how the field has developed further.

## Resolution of a cryo-tomogram

The raw data underlying a tomographic reconstruction are the projections collected in a TEM. Typically, data are acquired by tilting the sample around a single axis. Images are acquired at distinct tilt angles, which are changed linearly during tilt series acquisition with a constant tilt increment Δα. Assuming an approximately cylindrical object with diameter *D*, Crowther derived an estimate for the obtainable resolution, *r_C_*, of the 3D reconstruction (Crowther et al., 1970):

(1) $$r_{C}=\frac{1}{\Delta \alpha \times D}$$

**Fig. 1:**
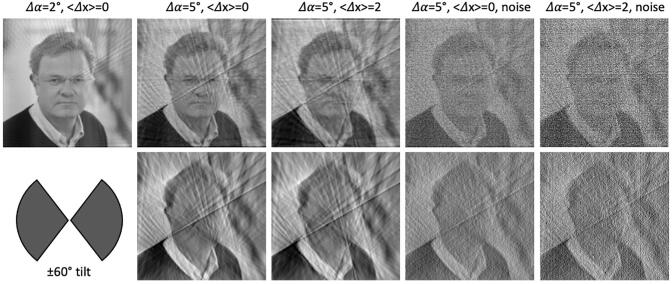
**Resolution of tomograms**. Top row: One dimensional projections of Wolfgang Baumeister have been computed with two different angular increments (*Δα* = 2° and *Δα* = 5°) and the two-dimensional image has been reconstructed using weighted backprojection. The top row displays the effect of the different *Δα* as well as the effect of alignment errors of the projection with a mean expected error, <*Δx*>, of 2 pixels, which is comparable to alignment errors typically obtained in tilt series alignment in cryo-ET. Moreover, white noise (SNR = 10, which is notably higher than that typically achieved in cryo tomograms) is added to the projections. Bottom row: the 2D image has been reconstructed from a limited tilt range of −60° to 60° as is common in cryo-ET, leaving a wedge-shaped region in Fourier space unsampled, as schematized by the symbol in the bottom left.

Importantly, this ‘Crowther theorem’ is a pure sampling consideration. If the tilt series had infinitely high SNR and the projection images were perfectly registered (see below), *r_C_* is the best possible resolution of the 3D reconstruction. For example, *r_C_* would correspond to approximately 1 nm detail level for a ribosome (D ∼ 25 nm) in a tilt series acquired with an increment of 2° (corresponding to ∼ 0.035 rad), which is unfortunately not achieved in raw cryo tomograms. Considering the sampling only, the level of trustworthy detail in a reconstruction increases with smaller angular increments and a smaller object is better resolved compared to a larger one with the same increment (Fig. 1).

A perennial challenge in cryo-ET is that reconstructions are not equally well resolved along all directions, even for perfectly aligned images with infinite SNR. To resolve different directions equally well the sample would need to be tilted from −90° to 90°, which is typically impossible due to the planar nature of the samples deposited on an EM grid. Most cryo-ET tilt series acquired today do not exceed −60° to 60°, leaving approximately 1/3 of the full 3D structure unsampled (Koster et al., 1997). In the Fourier transform of a reconstruction, these missing data give rise to a ‘missing wedge’, where these Fourier components are set to zero because they cannot be approximated reliably from the data. In real space, this missing wedge effect manifests as a strong directionality of the distinguishable level of detail. The resolution is highest perpendicular to the beam direction, while hardly any detail is distinguished along the beam direction (Fig. 1).

During acquisition of a tilt series the imaged part of the sample will move with respect to the tilt axis. To compensate for these motions the projection images need to be aligned (or ‘registered’) to a common coordinate system prior to reconstruction. Typically, an optimal coordinate system is found by a least squares optimization of 3D coordinates for gold markers that are added to the sample during the preparation (Lawrence, 1996). The relative positions of these gold markers are assumed to remain invariant throughout the acquisition of the tilt series and they define the coordinate system for the reconstruction. Similarly, the tilt series can be registered by more general ‘patches’ instead of fiducial markers that are tracked throughout the series, giving rise to the same linear equation system to be solved (Amat et al., 2010, Brandt et al., 2001). However, in both cases alignment inaccuracies are inevitable and, probably more importantly, it has become apparent that the frozen-hydrated sample is not entirely rigid throughout exposure (Campbell et al., 2012). Errors in tilt series registration result in blurring of the reconstruction and thereby limit the obtainable resolution (Fig. 1).

While the sampling and tilt series registration challenges could, in principle, be overcome by advanced engineering and computational methods, the beam sensitivity of the sample limits the obtainable resolution in a cryo-tomogram fundamentally. The electron beam alters the chemical structure of the sample with increasing dose. Typically, the applied doses are ∼ 100 electrons per Å^2^ in cryo-ET. Therefore, the signal-to-noise ratio (SNR) of the acquired tilt series is low. Since the intensity of structure factors of biological macromolecules decreases as a function of resolution the high-resolution signal is affected more strongly by the higher noise level, which limits the resolution (Rosenthal and Henderson, 2003). Typically, the level of detail that can be distinguished reliably in a raw cryo-tomogram rarely exceeds ∼ 5 nm when rigorous statistical measures, such as the Fourier Shell Correlation (FSC) (Saxton and Baumeister, 1982), are applied, even with today’s powerful detectors (Asano et al., 2015). Prior to the direct detector age when charge coupled devices (CCDs) or even films were used to record EM images, the resolution barely exceeded 10 nm.

## Early days of subtomogram averaging in Martinsried

Compared to X-rays, electrons are substantially less harmful to biological samples (Henderson, 1995). Nevertheless, X-rays were more popular than electrons in structural biology for decades because they could be applied to large crystals where the weak signal of millions of particles is coherently amplified. The idea underlying single particle analysis (SPA) is to align individual particles precisely in the computer such that their signals would also enhance each other in the resulting sum (Frank, 2017). In Martinsried, where Nobel laureate Joachim Frank started his scientific career under the mentorship of Walter Hoppe, it was soon realized that ET should be combined with particle alignment and averaging to obtain higher resolution insights into specific macromolecules. The first 3D reconstructions of the ribosomal 30S (Knauer et al., 1983) and 50S subunits (Oettl et al., 1983) were obtained by subtomogram averaging. The isolated, dried, and negatively stained subunits adopt preferred orientations on an EM grid, limiting the projections that could obtained and requiring the acquisition of tilt series to get clues about the full 3D structures. Alignment and averaging of ten 30S and five 50S particles notably improved the recognizable detail compared to the individual particles. Thus, subtomogram averaging relieved the resolution limitation due to the low SNR of the tilt series.

Wolfgang Baumeister continued the developments of Walter Hoppe on his quest to do structural biology in the cell. While the lower contrast of cryo-ET compared to ET of negatively stained particles was a challenge, the available computational power also increased continuously. For image processing a software package called EM was developed and used in Martinsried (Hegerl and Altbauer, 1982). The EM program provided a scripting language that was highly advanced at the time, though simple by today’s standards. It enabled simple coding of image processing algorithms and workflows that used well-tested built-in routines for basic tasks such as interpolations or Fourier transforms. This EM program allowed the implementation of correlation-based alignment and subsequent averaging of 100s of cryo-subtomograms (Walz et al., 1997). Like the early ribosome work, the main application of this algorithm was tomograms of isolated complexes that adopted preferred orientations on EM grids, which prevented the application of conventional SPA based on single projections of randomly oriented particles. This program provided the first morphological insights into the 26S proteasome (Walz et al., 1998) and it could even provide a pseudo-atomic model of a Hsp60 in its open conformation (Nitsch et al., 1998). This model of the Hsp60 thermosome in its open conformation, which was built by rigid-body fitting of the domains of the crystal structure of this complex in the closed conformation, was much later largely confirmed by a higher resolution cryo-EM SPA reconstruction (Zhang et al., 2010).

The development of subtomogram averaging in the EM software was an almost heroic achievement considering the restrictions of this software. The EM suite was programmed in FORTRAN 77, which made its usage awkward by today’s standards. For example, variables had fixed names ranging from ‘*?1*′ to ‘*?16*′ and arrays (e.g., images and volumes) were named ‘*?A*’ to ‘*?G*’, which made it rather difficult to keep an overview in programs and routines. The EM program had more constraints, but arguably the most irritating was the handling of usage errors that were bound to happen. After the first three errors, the EM program would encourage the user to operate with more care before terminating altogether after ten errors with the note “Bye, Bye, take a break”.

## AV3 package enables structural biology in native membranes

**Fig. 2:**
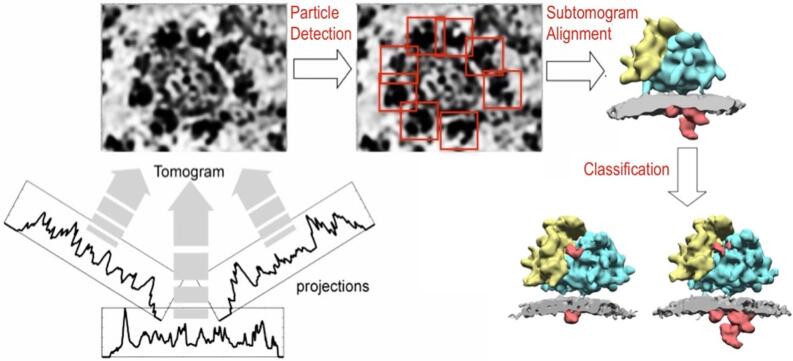
**Principle of subtomogram analysis explained for the ER-associated ribosome**. From the different projections acquired in the TEM, the tilt series, a tomographic volume is reconstructed, here a vesicle derived from the endoplasmic reticulum (ER). Subtomograms containing the particles of interest, in this example ribosomes (red boxes), are detected in the tomographic volume, and the subtomograms are rotationally and translationally aligned to a common coordinate system. In the resulting subtomogram average small (yellow) and large (cyan) ribosomal subunits, the lipid membrane (gray) and luminal protein density (red) are annotated. Subtomogram classification can then distinguish distinct states of the complex of interest. In this example, the ER translocon sub-complex oligosaccharyltransferase is present or absent in the two classes. (For interpretation of the references to colour in this figure legend, the reader is referred to the web version of this article.)

When cryo-tomograms had become easier to acquire the demands on image processing also increased and it was evident that contemporary programming and scripting languages allowed much faster realization of new algorithms. Thus, PhD students and postdocs in Wolfgang Baumeister’s laboratory in Martinsried teamed up to compile the TOM toolbox, which ported the main functionality of the EM program to the commercial scripting language MATLAB (Nickell et al., 2005). This toolbox allowed the development of a new workflow for subtomogram averaging (Förster et al., 2005). The resulting AV3 package is still available and maintained to some extent (https://bitbucket.org/FridoF/av3/) with its main features explained in more detail in the appendix. The AV3 package, as well as its successor PyTom (Hrabe et al., 2012) and many other subtomogram analysis packages, enable the workflow depicted in Fig. 2. Tomograms are reconstructed from multiple tilt series, subtomograms depicting particles of interest are detected, they are aligned to a common coordinate system, averaged, and in some cases classified further into different molecular states.

Similar to the previously established algorithm by Walz et al (Walz et al., 1997), the AV3 package uses a quasi-expectation maximization algorithm to align subtomograms such that the resulting average is most similar to the input data. A major difference of the AV3 algorithms was the similarity measure, namely a new constrained correlation function that accounted for the missing wedge effect. This missing wedge filter turned out to be highly efficient in increasing the alignment accuracy for anisotropic structures. Important membrane-associated complexes, which became a major interest of mine and some of my colleagues, fall into this category.

Conventional cryo-EM SPA was only applicable to isolated macromolecules, which required solubilization in the case of membrane proteins and their complexes. Cryo-ET now provided the possibility of imaging membrane-associated complexes in their native lipid environments, obviating the need of solubilization and thereby avoiding potential structural alterations by detergents. Personally, I was interested in resolving the structures of retroviral envelope protein complexes (Env) in their viral membrane, while my fellow PhD student Martin Beck and Ohad Medalia had the ambitious aim to get some insight into a truly gigantic membrane-associated protein complex, the Nuclear Pore Complex (NPC). These two projects powered the development of a pipeline for subtomogram averaging of particles located on virions and on isolated nuclei. Initially, the particles were located manually on their respective membranes with appropriate interactive tools. The subsequent iterative averaging protocol required good initial approximations of the particle orientations because only a fraction of the rotational space could be searched. In the case of membrane-associated complexes two of three Euler angles can be directly approximated well due to their invariant orientation with respect to their embedding membrane, which proved sufficient for successful convergence in our projects. Importantly, we could also determine the rotational symmetry of membrane-associated particles, which was then used to further improve the resolution of the subtomogram averages (Förster and Hegerl, 2007).

Our initial efforts went into the structure determination of the NPC (Beck et al., 2007, Beck et al., 2004), for which the recording of every tilt series and subsequent localization of NPCs in the tomograms was a considerable achievement due to the thickness of the sample. Even at its periphery, the thickness of the nuclei approached 1 μm rapidly, which resulted in an extremely low SNR of the acquired tilt series. Nevertheless, due to the extremely high molecular mass of NPCs (ranging from 60 MDa in yeast to over 120 MDa in vertebrates) we succeeded in obtaining an 8 nm resolution reconstruction, which provided by far the most detailed insights into the NPC at the time. Structural characterization of the Env of murine leukemia virus using the same methodology achieved a much higher resolution because the sample’s diameter was only ∼100 nm and the particles were much more abundant in tomograms compared to NPCs (Förster et al., 2005). These subtomogram averages exceeded 3 nm resolution, coinciding with the first oscillation of the contrast-transfer function (a function relating the observed intensities for different spatial frequencies to the applied defocus), which we did not compensate for in our experiments. This resolution level even allowed fitting atomic models of Env fragments into the map.

The concept of a constrained correlation was later extended for the purpose of subtomogram classification, i.e., the grouping of subtomograms according to different molecular states (Förster et al., 2008), completing the workflow of subtomogram analysis needed to tackle structurally heterogeneous particle populations (Fig. 2). The AV3 program was useful for many subsequent studies in Martinsried, but it also served its purpose outside the lab. For example, the package allowed reconstruction of the SIV Env trimer (Zanetti et al., 2006). The Briggs lab modified the suite, e.g., to include contrast transfer function correction (Zanetti et al., 2009), and used it very successfully in many subsequent subtomogram averaging studies of viral protein complexes and vesicle coats (Briggs, 2013). The package also inspired the development of similar, mostly matlab-based packages such as PEET (Nicastro et al., 2005) and Dynamo (Castano-Diez et al., 2012). In my own research, I mostly converted to a new software package PyTom, which we based on open-source software and maintains most of the functionality of AV3 (Hrabe et al., 2012). This platform is suitable for large-scale computations and enables integration of many open-source tools such as spherical harmonics expansion for exhaustive rotation sampling (Chen et al., 2013, Chen et al., 2014) and iterative Fourier reconstruction methods (Chen and Förster, 2014).

The abovementioned programs for subtomogram averaging and their classification all employ binary assignments of orientation and classes. In cryo-EM SPA maximum likelihood (ML) or maximum a posteriori (MAP) approaches such as implemented in the software RELION are preferred (Scheres, 2012). In Martinsried, ML approaches to subtomogram alignment and classification with ‘compound weighting’ of the missing wedge have been developed early on (Stölken et al., 2010). Despite the encouraging results, the computational effort of ML approaches has been prohibitive for routine application in cryo-ET at the time, but this situation has changed meanwhile (Bharat and Scheres, 2016).

## Structural biology in the cellular context and template matching

Structural biology in the cell using cryo-ET entails comprehensive mapping of macromolecules in the field of view and subsequently analyzing their detailed structure(s) using subtomogram analysis. Wolfgang Baumeister’s group pursued the development of template matching as an approach to systematically map macromolecules of known structure (Böhm et al., 2000). In this approach, the previously determined structure of the molecule of interest serves as a template, which is rotated into different orientations and compared with the tomogram voxel by voxel using a correlation function. Again, constraining the correlation to the sampled part of the data (thus accounting for the missing wedge effect) notably improves the sensitivity and specificity of the approach. In a proof-of-concept study using liposomes with defined macromolecular content the feasibility of high-performance detection of large (>700 kDa) complexes could be demonstrated (Frangakis et al., 2002). Of note, template matching is not restricted to distinct macromolecules and can also be extended to filamentous structures, which are identified based on shorter, effectively cylindrical segments (Rigort et al., 2012).

The emergence of direct detectors has further propelled the application of comprehensive template matching and subtomogram analysis in the cellular context. For example, 26S proteasomes, the major intracellular protease in eukaryotic cells and arguably Wolfgang Baumeister’s favorite macromolecule, could be exhaustively mapped in neurons (Asano et al., 2015). Subtomogram analysis then revealed the relative distributions of single versus double-capped proteasomes and even nucleotide-dependent conformational changes. In subsequent studies, the detailed molecular impact of different types of amyloid aggregations on 26S proteasomes could be studied in cellular systems mimicking aspects of different neurodegenerative diseases (Guo et al., 2018, Riemenschneider et al., 2021) . Thus, the vision of structural biology *in situ* has already come to fruition for large complexes such as the ∼2MDa 26S proteasome.

While template matching can be a great help to structurally mine cryo tomograms it is ultimately preferred to analyze the data without external hypotheses that may bias results. The challenge are the vast volumes to be analyzed. Martinez-Sanchez et al have developed a template-free approach that segments lipid membranes in tomograms and automatically classifies associated densities (Martinez-Sanchez et al., 2020). Such template-free approaches will become more common in future mapping of macromolecules in the cell.

## Merging of subtomogram and single particle analysis

Subtomogram averaging, as described above, overcomes the resolution limitations due to the limited electron dose and angular sampling of the tilt series, but it does not address the limits arising from the limited accuracy of tilt series registration (Fig. 1). The phenomena of beam-induced motion and radiation damage mean that vitreous samples remain neither stationary nor invariant during beam exposure (Brilot et al., 2012). Accordingly, global registration of a tilt series will always have limited accuracy. In cryo-EM SPA, the reconstruction is obtained directly from the projections of the individual particles, which are aligned independently without the context of their variable surroundings. Thus, SPA does not involve an intermediate step via a blurred subtomogram. In other words, SPA faces the challenge of registering projections only locally, which is less affected by beam-induced motion than global registration of tilt series. Applying SPA-like approaches directly to the tilt series would thus allow aligning the depicted particles more precisely. One challenge in this approach is that images of a tilt series are typically acquired with only 1–2 e^-^/Å^2^, which is less than a tenth of the dose that is typically used to acquire a micrograph for SPA. Another, fundamental consideration is that SPA requires the particles of interest to be isolated, both to avoid extensive classifications and overlapping projections. This condition is not fulfilled in cellular cryo-ET, where any projection of a particle will also contain the signal from other molecules in the projection path, possibly even from another copy of the same type of molecule. Thus, the question became whether this violation of the isolated object is tolerable for thin samples and how SPA-like alignment would deal with the extremely low SNR of the tilt series.

**Fig. 3:**
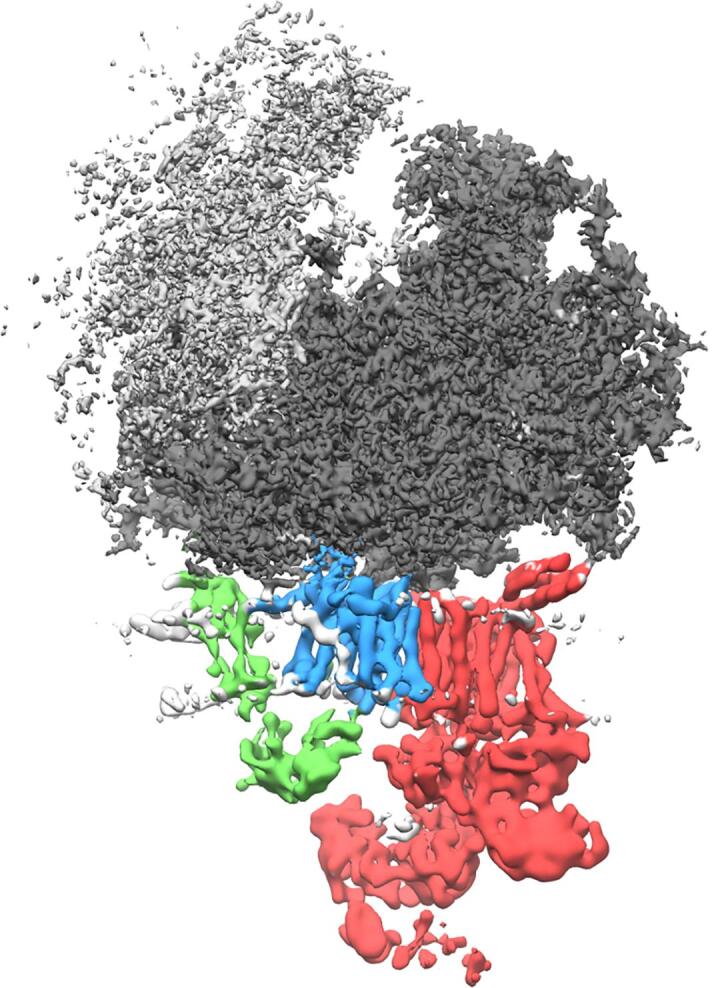
**High-resolution reconstruction of the ER-associated ribosome**. A reconstruction of the ribosome bound to the ER translocon in its native membrane has been obtained from tomograms of ER-derived vesicles (Gemmer et al, unpublished). Compared to previous studies (Braunger et al., 2018, Pfeffer et al., 2015) the resolution has been improved substantially through local alignment of ribosome-translocon particles using the software M. The ribosome (40S subunit light gray, 60S subunit dark gray) is filtered to 4 Å and the membrane-embedded ER translocon subunits TRAP (green), Sec61 (blue) and oligosaccharyl transferase (red) to 8 Å. (For interpretation of the references to colour in this figure legend, the reader is referred to the web version of this article.)

For a tilt series of biochemically isolated particles such as ribosomes, the application of SPA-like approaches yielded notable improvements in resolution compared to subtomogram averaging (Chen et al., 2019, Himes and Zhang, 2018). Key to success of such approaches was an efficient regularization of particle alignments within the vicinity of their positions and orientations determined in the tomograms. Due to the extremely low SNRs of the tilt series images the particles are prone to misalignment otherwise. A particularly successful regularization was developed by Dimitry Tegunov, who became interested in formulating the problem of image and particle alignment as an optimization problem already as a student assistant in Martinsried (Tegunov et al., 2021). The program M, which he later developed during his PhD at the Max-Planck Institute in Göttingen, regularizes the 3D positions of particles in a tomogram throughout data acquisition in the form of splines. Thus, instead of using many particle positions, a significantly smaller number of spline parameters characterizes the sample deformation, strongly reducing the degree of overfitting for noisy data. The program interfaces with the popular SPA software RELION (Scheres, 2012), which allows usage of its well-developed CTF correction and classification methods in cryo-ET (Bharat and Scheres, 2016). This combination of M with RELION enabled the localization of ribosome-bound antibiotics in the context of very thin bacterial cells (Tegunov et al., 2021). Importantly, this workflow is readily reproducible (Fig. 3), suggesting that for large complexes in thin samples the number of collected particles eventually limits the obtainable resolution. At this point, the application of SPA methodology to tilt series has succeeded in reaching the regime of ∼5 Å or better only for ribosomes, which are comparably rewarding targets due to their high molecular mass and strong contrast resulting from their high RNA content. What the limits of such SPA approaches are in terms of molecular mass and density of the complex of interest and sample thickness when applied to tilt series data will be important to evaluate.

## Concluding remarks

Computation has always been an integral component of cryo-ET. Artificial intelligence has impacted computation profoundly, including structural biology. AlphaFold structure predictions of individual proteins often appear almost indistinguishable from experimental structures based on X-ray diffraction and cryo-EM data (Jumper et al., 2021). Thus, the field of structural biology is in transition and its focus will move away even further from individual components towards the interaction of macromolecules in their physiological environment. Carol Robinson, Andrej Šali and Wolfgang Baumeister have eloquently termed this discipline as the elucidation of the ‘molecular sociology of cells’ (Robinson et al., 2007). The recent advances in subtomogram averaging combined with the developing impact of deep learning approaches on particle localization in cryo tomograms (Gubins et al., 2020) and 3D reconstruction (Liu et al., 2021) suggest that cryo-ET will play a key role in putting the puzzle pieces provided by AlphaFold into cellular context. The unique capability of cryo-ET to image the native state will be invaluable to elucidate the largely uncharted territories of organelles and membranes, which are often regulated by low-affinity interactions that are inaccessible in a test tube.

## Declaration of Competing Interest

The authors declare the following financial interests/personal relationships which may be considered as potential competing interests:

Friedrich Förster reports financial support was provided by European Research Council. Friedrich Förster reports financial support was provided by Nederlandse Organisatie voor Wetenschappelijk Onderzoek Utrecht.

## Appendix

The AV3 package uses an iterative quasi expectation–maximization algorithm to coherently align subtomograms and average them. The aim of the algorithm is to maximize the similarity of appropriately rotated and translated subtomograms *P_i_* to an iteratively updated reference *R*. A major feature of the AV3 package is the similarity measure that underlies the algorithm, termed the constrained correlation function *CCF*. The idea of the *CCF* is to restrict the comparison of *P_i_* and *R* to the areas that are sampled in both, i.e., to exclude the missing wedge in Fourier space from the score. Thus, the missing-wedge-filtered reference would be:

(2) $$\overset{\;}{R}={\mathrm{FT}}^{-1}\left(W\cdot FT(R)\right)$$

Here, *FT* denotes the Fourier transform, *FT^−1^* its inverse and *W* is a filter specifying the weight (i.e., it would be zero inside the missing wedge). When *R* is rotated (*Rot*) by Euler angles (*φ,θ,ψ*) and translated (*Trans*) by *(Δx,Δy,Δz*) the transformed and filtered reference is:

(3) $${\overset{\;}{R}}_{\phi ,\theta ,\psi ,\Delta x,\Delta y,\Delta z}={\mathrm{Trans}}_{\phi ,\theta ,\psi }\left({\mathrm{FT}}^{-1}\left(W\cdot FT({\mathrm{Rot}}_{\phi ,\theta ,\psi }\left(R\right))\right)\right)$$

With this definition the Pearson correlation function of the transformed and filtered reference with the filtered subtomogram becomes:

(4) $${\mathrm{CCF}}_{\phi ,\theta ,\psi ,\Delta x,\Delta y,\Delta z}=\frac{\sum_{v=1}^{N_{v}}{\left({\overset{\;}{R}}_{\phi ,\theta ,\psi ,\Delta x,\Delta y,\Delta z}-\overset{-}{\overset{\;}{R}}\right)}_{\nu }\cdot {\left({\overset{\;}{P}}_{i}-\overset{-}{{\overset{\;}{P}}_{i}}\right)}_{\nu }}{\sqrt{\sum_{v=1}^{N_{v}}{\left({\overset{\;}{R}}_{\phi ,\theta ,\psi ,\Delta x,\Delta y,\Delta z}-\overset{-}{\overset{\;}{R}}\right)}_{\nu }^{2}\cdot {\left({\overset{\;}{P}}_{i}-\overset{-}{{\overset{\;}{P}}_{i}}\right)}_{\nu }^{2}}}$$

Here, ν is the index of the voxel, N_ν_ the number of voxels in subtomogram and reference, and the bar indicates the mean value.

The main benefit of the filtering in equation (4) is the normalization, which proves beneficial for structures with anisotropic distribution of their signal in Fourier space. For example, membrane-associated complexes fall into this category, which are so interesting to explore using cryo-ET.

The *CCF* is in the core of a simple algorithm to determine the density *R* that is most consistent with the subtomograms *P_i_*. In the (quasi) expectation step ${\mathrm{CCF}}_{\phi ,\theta ,\psi ,\Delta x,\Delta y,\Delta z}$ is computed in a six-dimensional search. While the translational parameters can be scanned exhaustively using Fourier transforms, the rotational search is restricted to the neighborhood of an initial seed. Exhaustive rotational search can be achieved via a later-developed spherical harmonics implementation (Chen et al., 2013). Each *P_i_* is assigned the orientation and translation that yielded the highest *CCF*, which is then used to generate *R* for the next iteration by averaging the inversely transformed *P_i_* in the maximization step. Importantly, this averaging procedure also includes wedge weighting.
